# Supplementary material for: Intelligent auxiliary system for music performance under edge computing and long short-term recurrent neural networks
Source: PLoS One. 2023 May 8;18(5):e0285496. doi: 10.1371/journal.pone.0285496 (PMC10166492; doi:10.1371/journal.pone.0285496)
Supplement: S1 Data — (ZIP) [file pone.0285496.s001.zip › data/figure 11.pptx]

## Slide 1
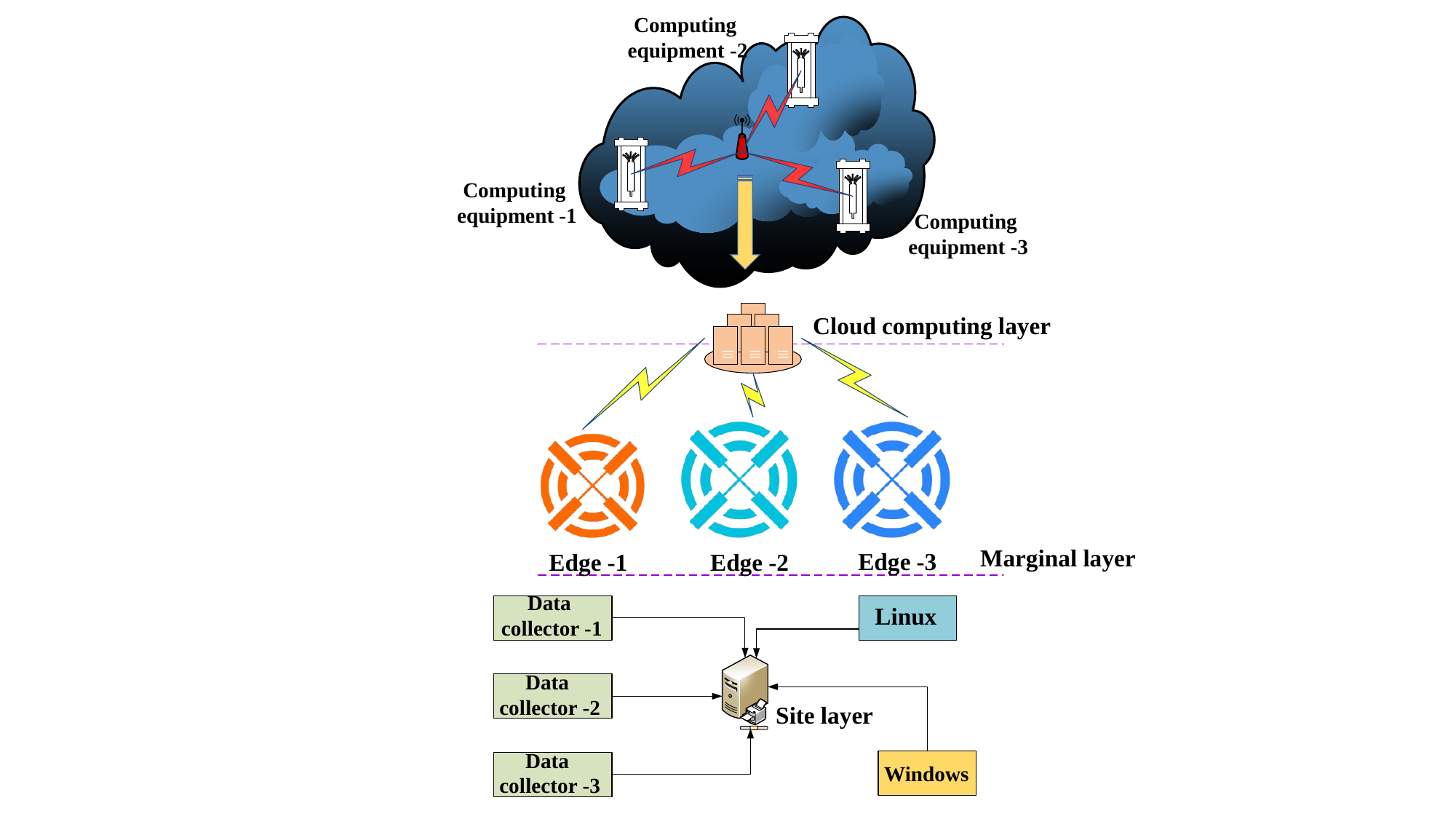

Computing
equipment -2
Computing
equipment -1
Computing
equipment -3
Cloud computing layer
Marginal layer
Edge -3
Edge -1
Edge -2
Data
collector -1
Linux
Data
collector -2
Site layer
Data
collector -3
Windows
