# Supplementary material for: Intelligent auxiliary system for music performance under edge computing and long short-term recurrent neural networks
Source: PLoS One. 2023 May 8;18(5):e0285496. doi: 10.1371/journal.pone.0285496 (PMC10166492; doi:10.1371/journal.pone.0285496)
Supplement: S1 Data — (ZIP) [file pone.0285496.s001.zip › data/figure 12.pptx]

## Slide 1
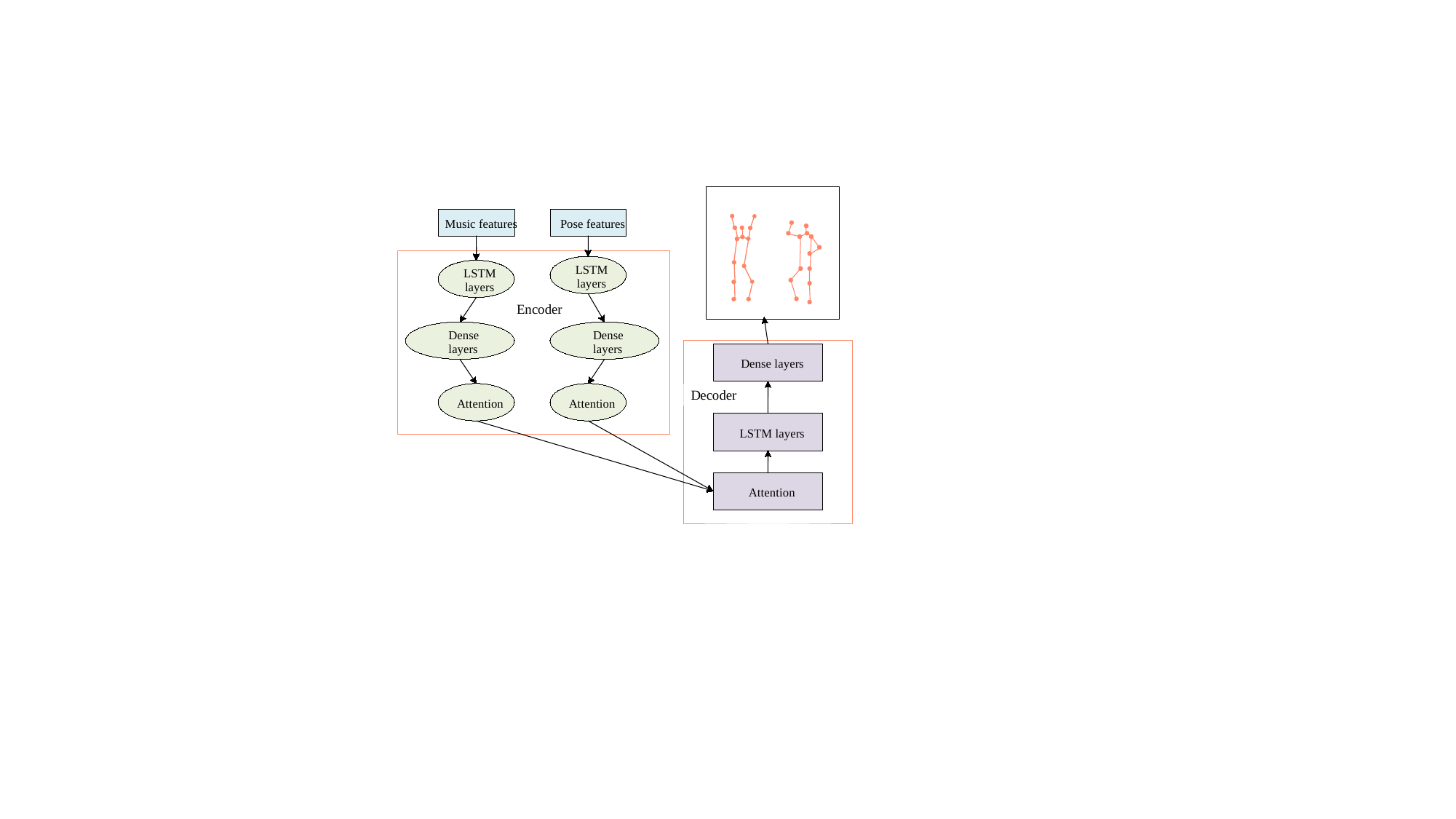

Music features
Pose features
LSTM
LSTM
layers
layers
Encoder
Dense
Dense
layers
layers
Dense layers
D
ecoder
Attention
Attention
LSTM layers
Attention
