# Supplementary material for: Intelligent auxiliary system for music performance under edge computing and long short-term recurrent neural networks
Source: PLoS One. 2023 May 8;18(5):e0285496. doi: 10.1371/journal.pone.0285496 (PMC10166492; doi:10.1371/journal.pone.0285496)
Supplement: S1 Data — (ZIP) [file pone.0285496.s001.zip › data/figure 15.pptx]

## Slide 1
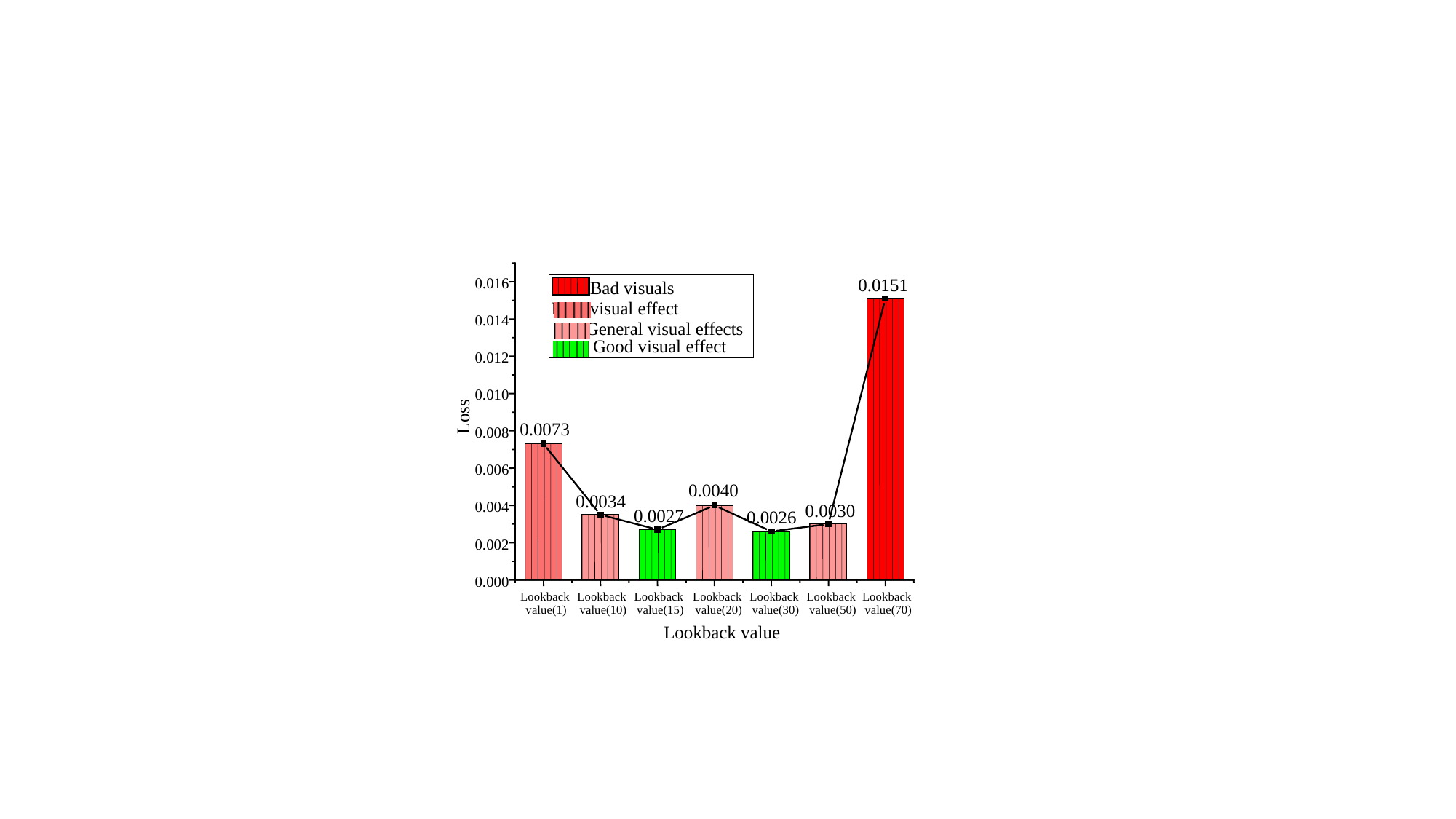

0.016
 Bad visuals
 Poor visual effect
0.014
General visual effects
Good visual effect
0.012
0.010
Loss
0.008
0.006
0.004
0.002
0.000
Lookback
Lookback
Lookback
Lookback
Lookback
Lookback
Lookback
value(1)
value(10)
value(15)
 value(20)
 value(30)
 value(50)
value(70)
Lookback value
0.0151
0.0073
0.0040
0.0034
0.0030
0.0027
0.0026
