# Supplementary material for: Intelligent auxiliary system for music performance under edge computing and long short-term recurrent neural networks
Source: PLoS One. 2023 May 8;18(5):e0285496. doi: 10.1371/journal.pone.0285496 (PMC10166492; doi:10.1371/journal.pone.0285496)
Supplement: S1 Data — (ZIP) [file pone.0285496.s001.zip › data/figure 16.pptx]

## Slide 1
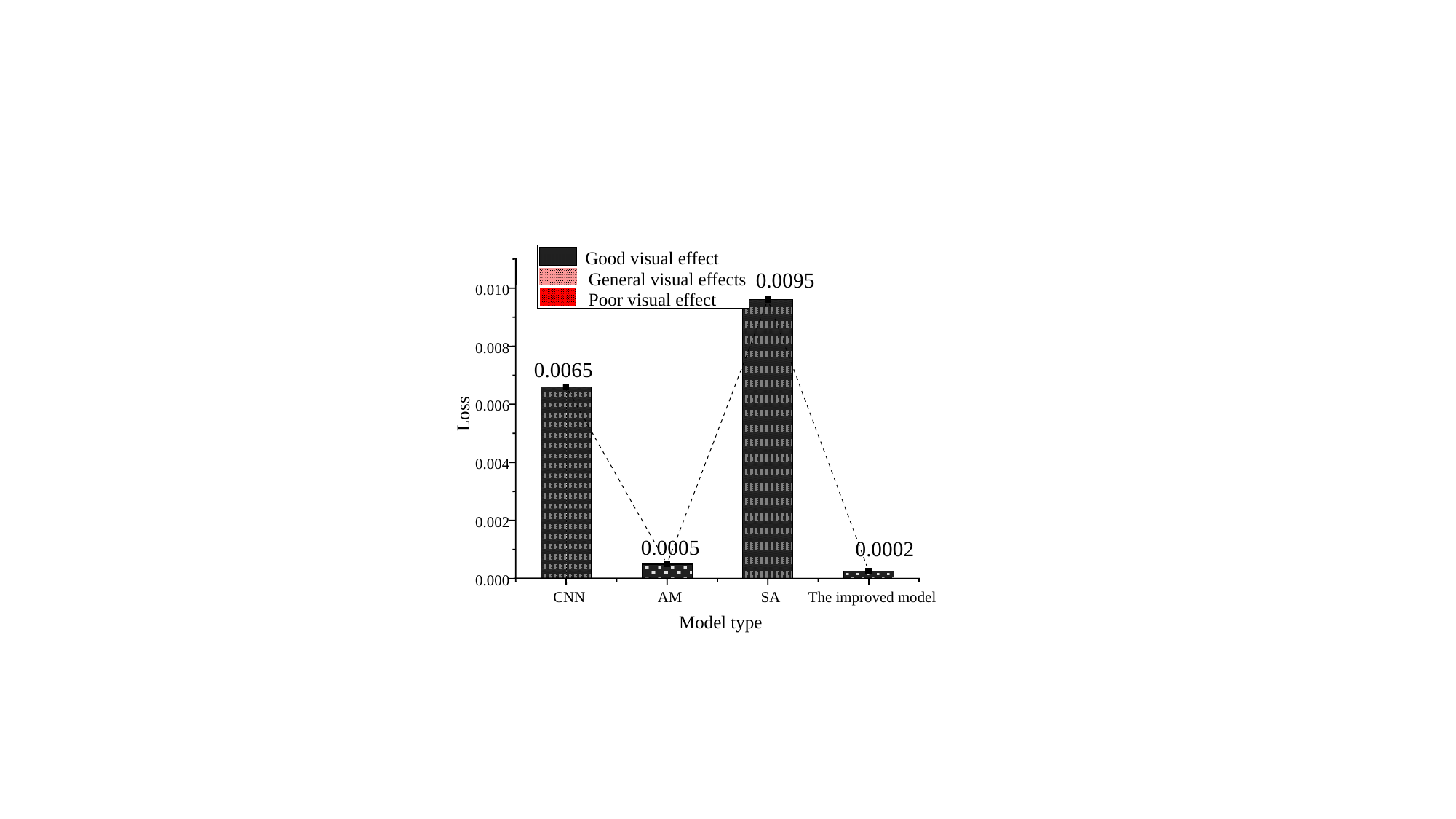

Good visual effect
 General visual effects
0.010
 Poor visual effect
0.008
0.006
Loss
0.004
0.002
0.000
CNN
AM
SA
The improved model
Model type
0.0095
0.0065
0.0005
0.0002
