# Supplementary material for: Intelligent auxiliary system for music performance under edge computing and long short-term recurrent neural networks
Source: PLoS One. 2023 May 8;18(5):e0285496. doi: 10.1371/journal.pone.0285496 (PMC10166492; doi:10.1371/journal.pone.0285496)
Supplement: S1 Data — (ZIP) [file pone.0285496.s001.zip › data/figure 8.pptx]

## Slide 1
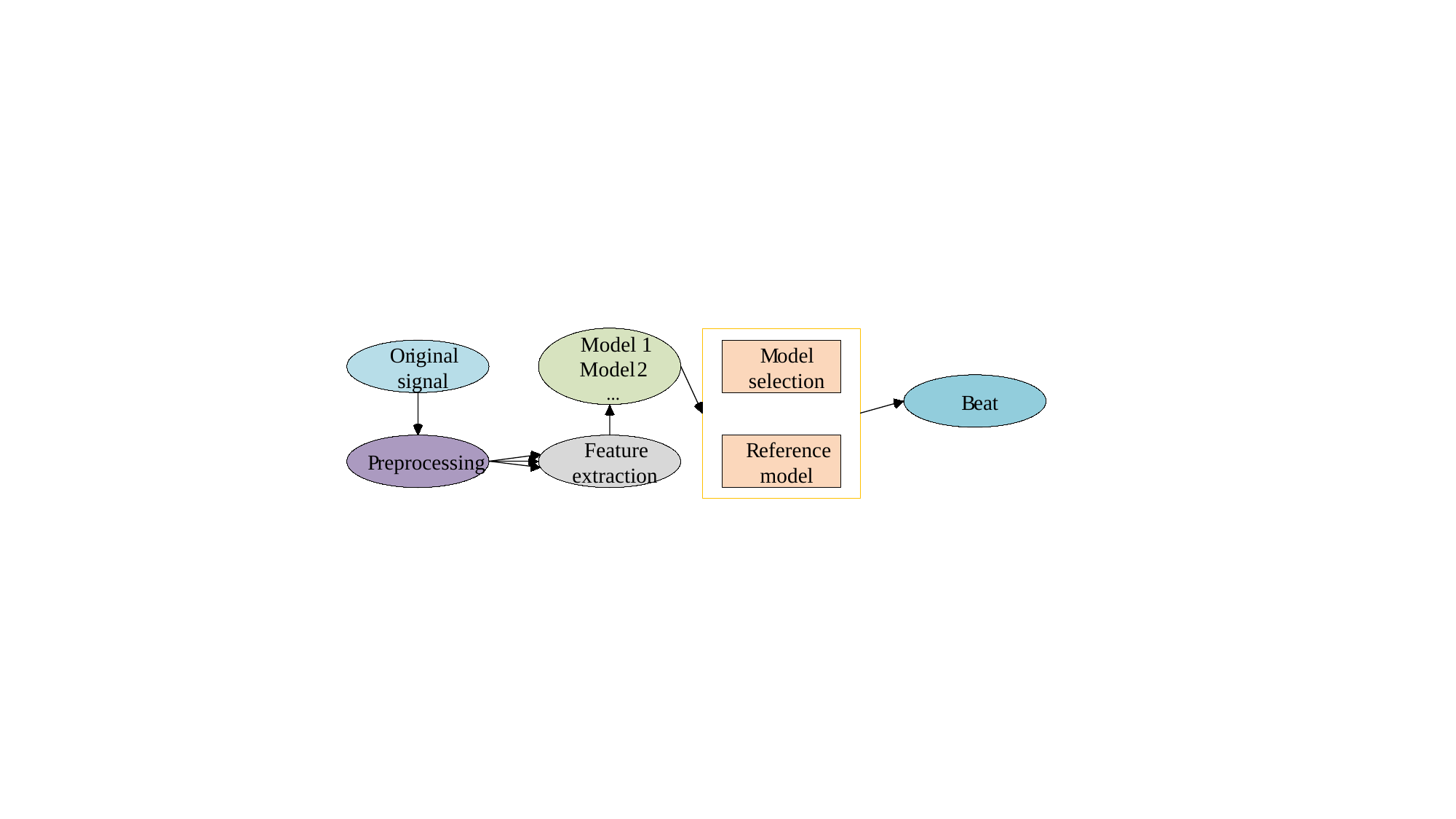

Model 1
Or
iginal
M
odel
Model
2
signal
selection
...
B
eat
Feature
R
eference
P
reprocessing
extraction
model
