# Supplementary figures and images for: Intelligent auxiliary system for music performance under edge computing and long short-term recurrent neural networks
Source: PLoS One. 2023 May 8;18(5):e0285496. doi: 10.1371/journal.pone.0285496 (PMC10166492; doi:10.1371/journal.pone.0285496)

## Slide 1
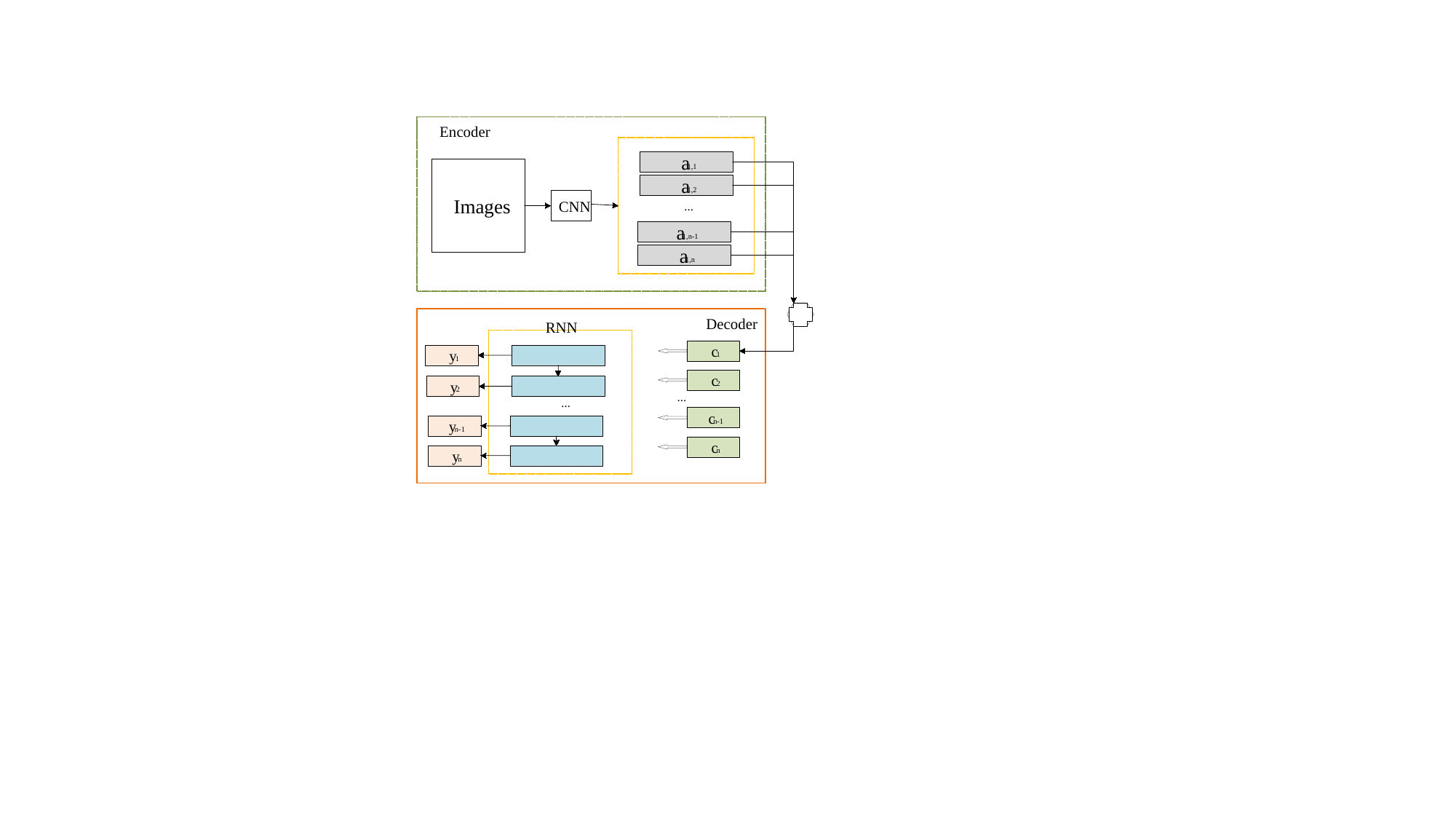

Encoder
a
1,1
a
1,2
Images
CNN
...
a
1,n
-
1
a
1,n
D
ecoder
RNN
c
y
1
1
c
y
2
2
...
...
c
n
-
1
y
n
-
1
c
n
y
n

Supplement: S1 Data — (ZIP) [file pone.0285496.s001.zip › data/figure 1.pptx]

## Slide 1
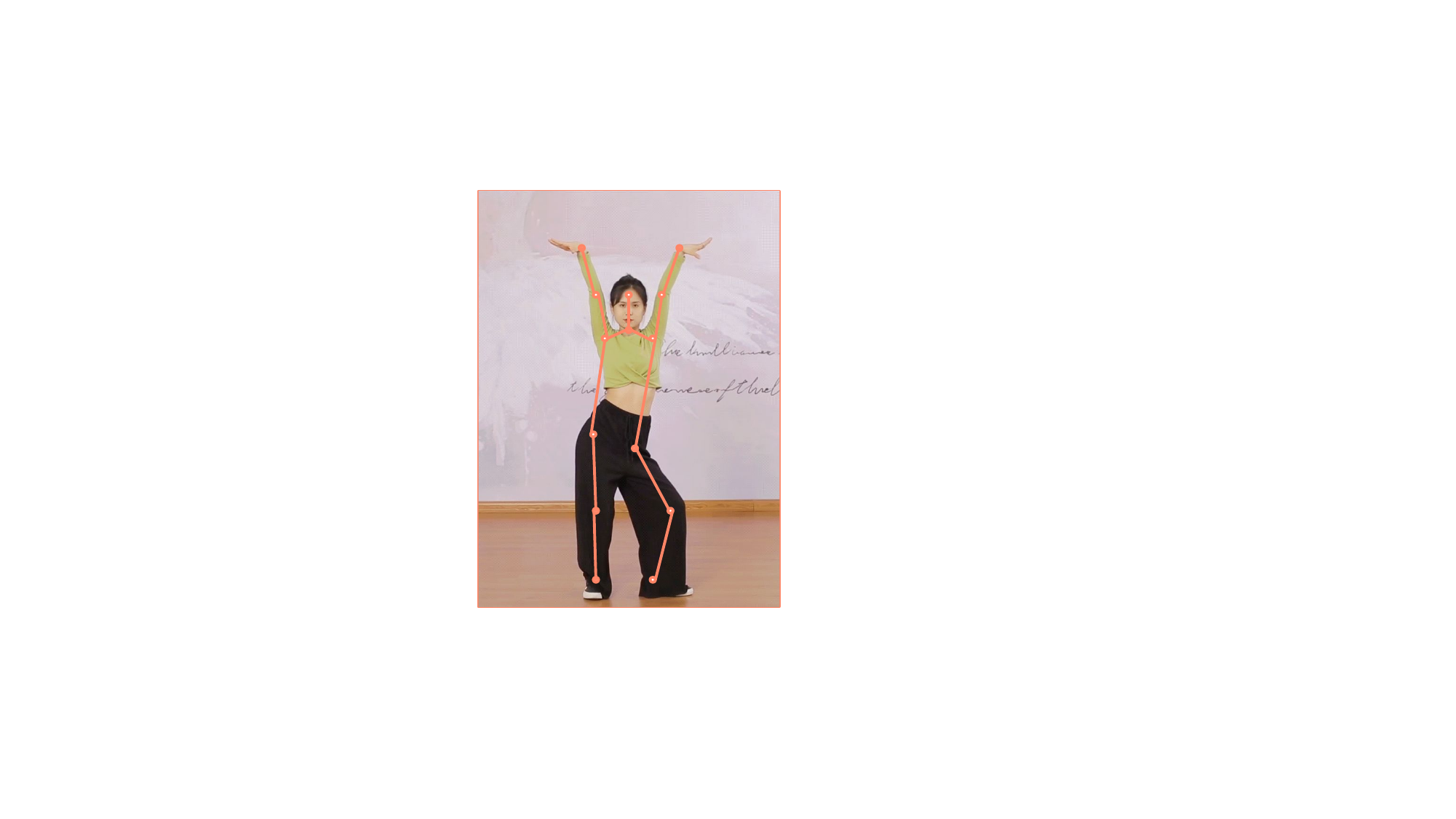

Supplement: S1 Data — (ZIP) [file pone.0285496.s001.zip › data/figure 10.pptx]

## Slide 1
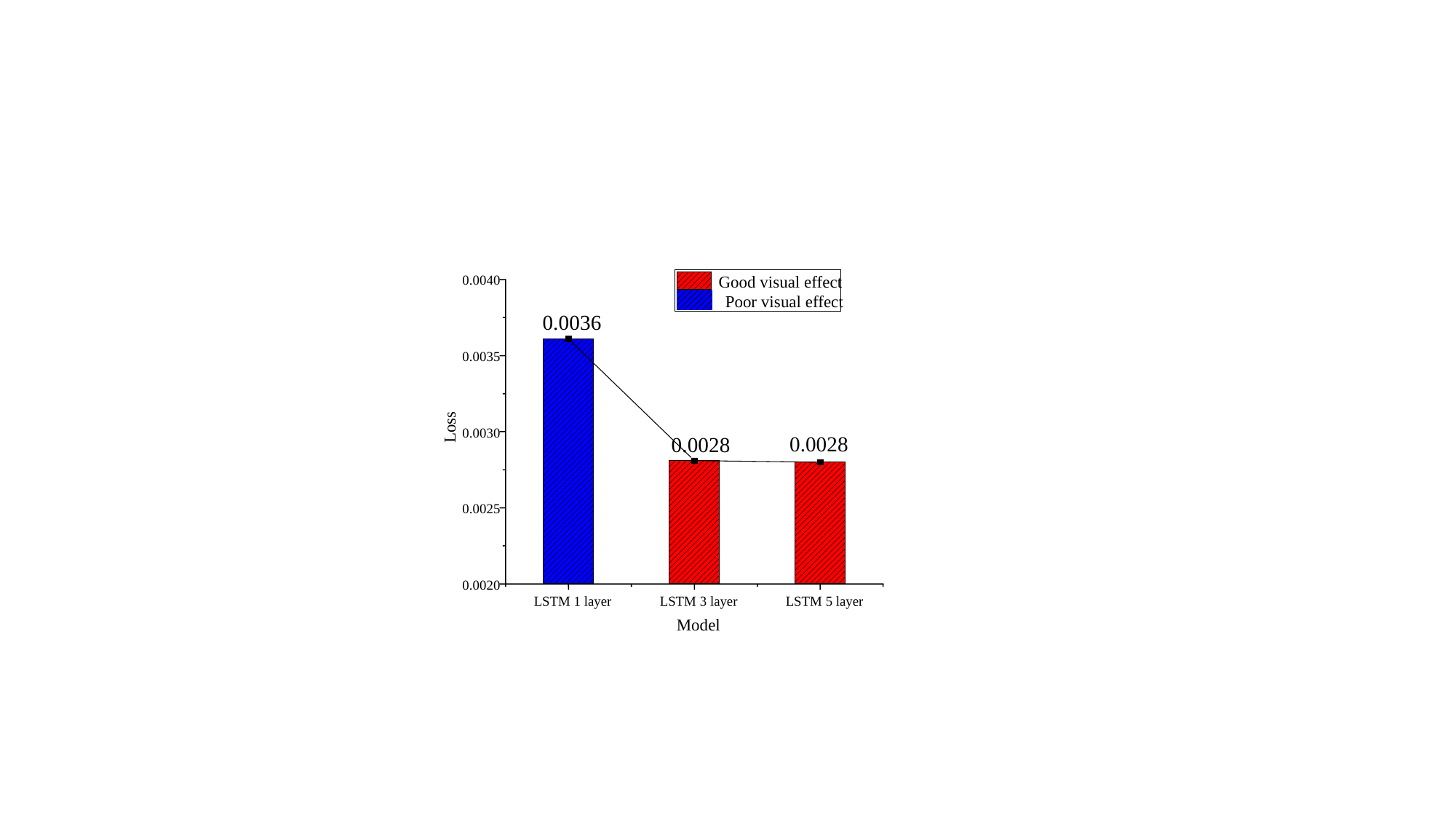

Good visual effect
0.0040
 Poor visual effect
0.0035
Loss
0.0030
0.0025
0.0020
LSTM 1 layer
LSTM 3 layer
LSTM 5 layer
Model
0.0036
0.0028
0.0028

Supplement: S1 Data — (ZIP) [file pone.0285496.s001.zip › data/figure 13.pptx]

## Slide 1
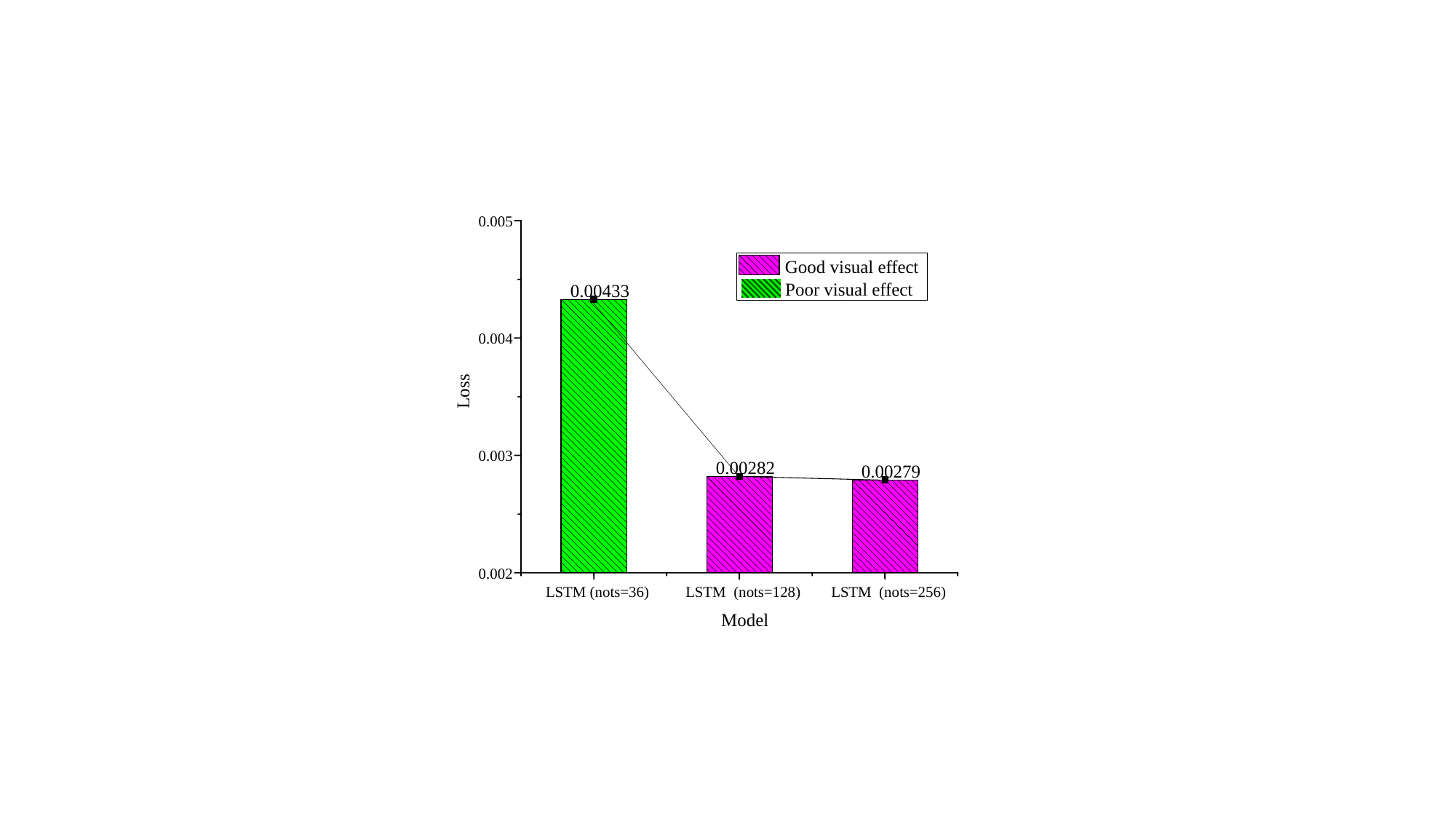

0.005
 Good visual effect
 Poor visual effect
0.00433
0.004
Loss
0.003
0.00282
0.00279
0.002
LSTM (nots=36)
LSTM (nots=128)
LSTM (nots=256)
Model

Supplement: S1 Data — (ZIP) [file pone.0285496.s001.zip › data/figure 14.pptx]

## Slide 1
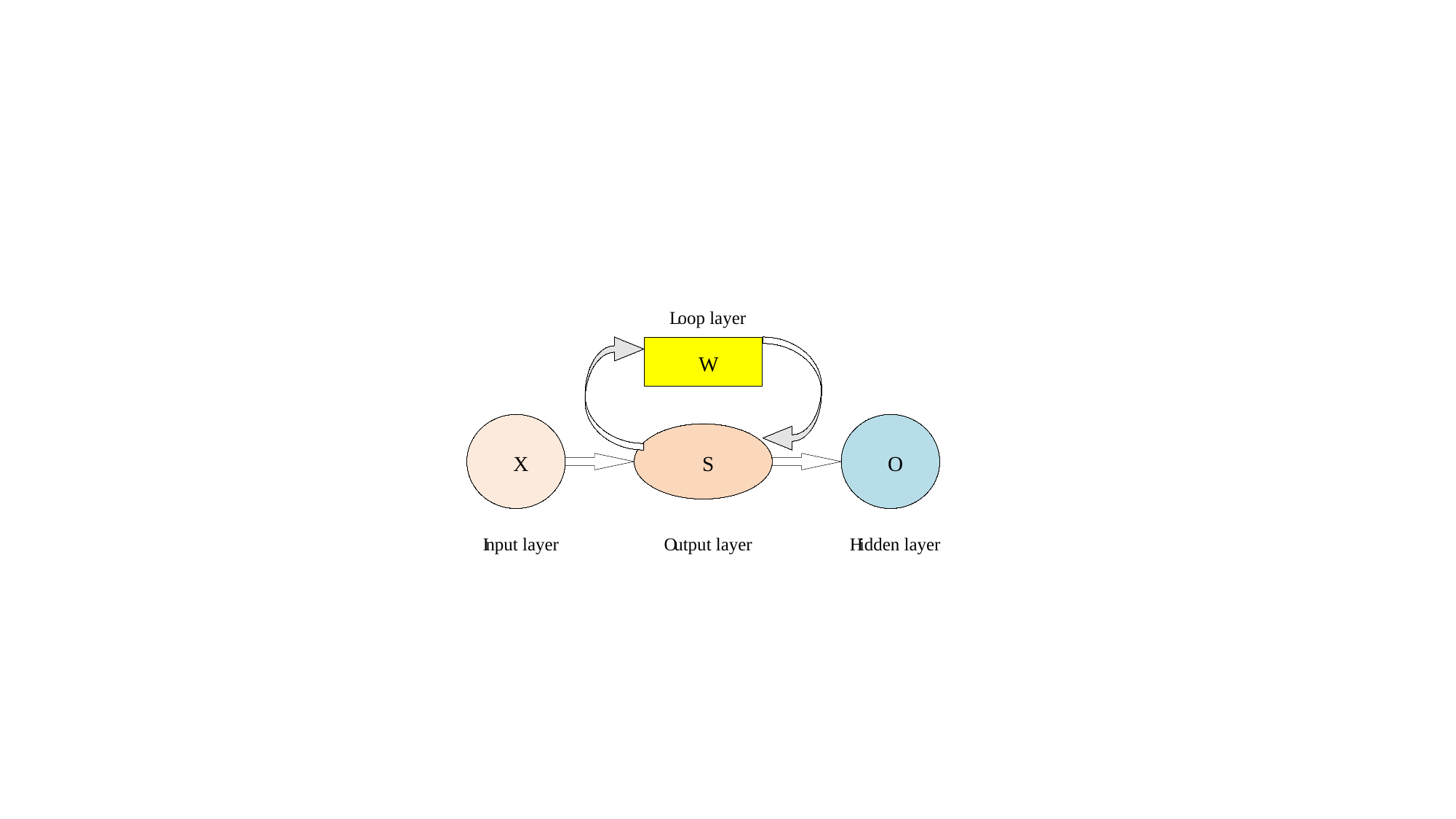

L
oop layer
W
X
S
O
I
nput layer
O
utput layer
H
idden layer

Supplement: S1 Data — (ZIP) [file pone.0285496.s001.zip › data/figure 2.pptx]

## Slide 1
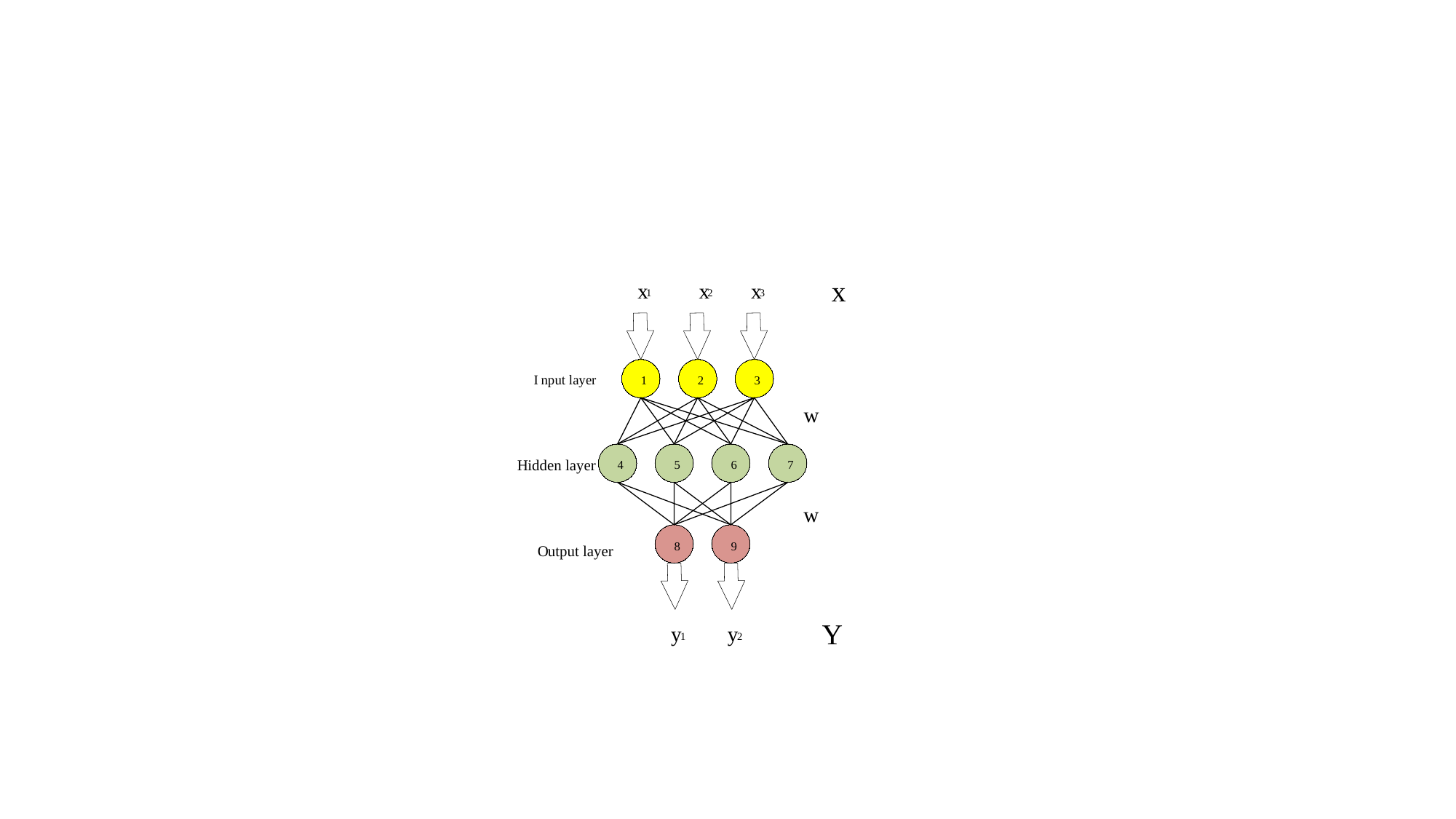

x
x
x
x
1
2
3
I
nput layer
1
2
3
w
H
idden layer
4
5
6
7
w
8
9
O
utput layer
Y
y
y
1
2

Supplement: S1 Data — (ZIP) [file pone.0285496.s001.zip › data/figure 3.pptx]

## Slide 1
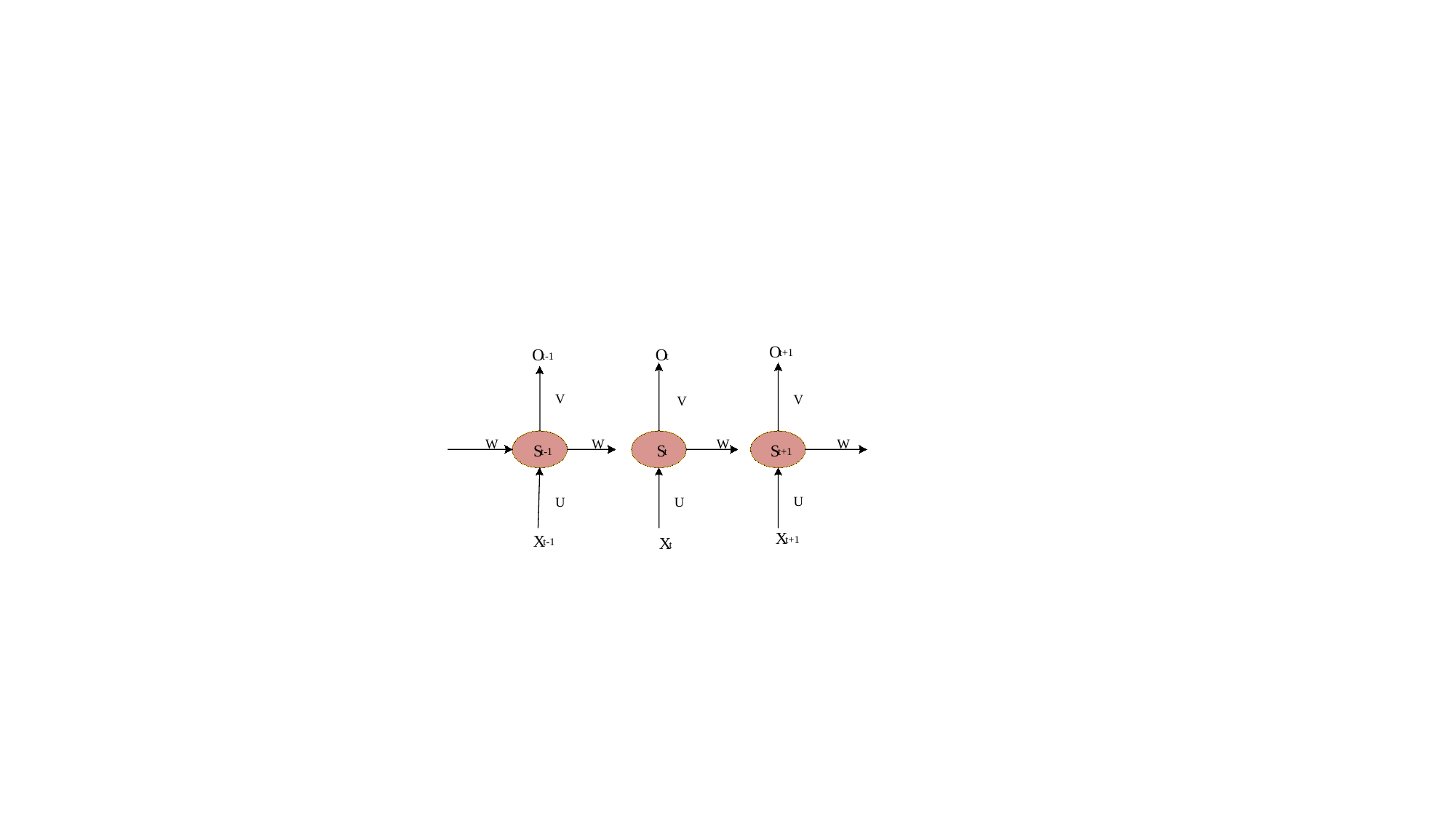

O
O
O
t+1
t
-
1
t
V
V
V
W
W
W
W
S
S
S
t
-
1
t
t+1
U
U
U
X
X
t+1
X
t
-
1
t

Supplement: S1 Data — (ZIP) [file pone.0285496.s001.zip › data/figure 4.pptx]

## Slide 1
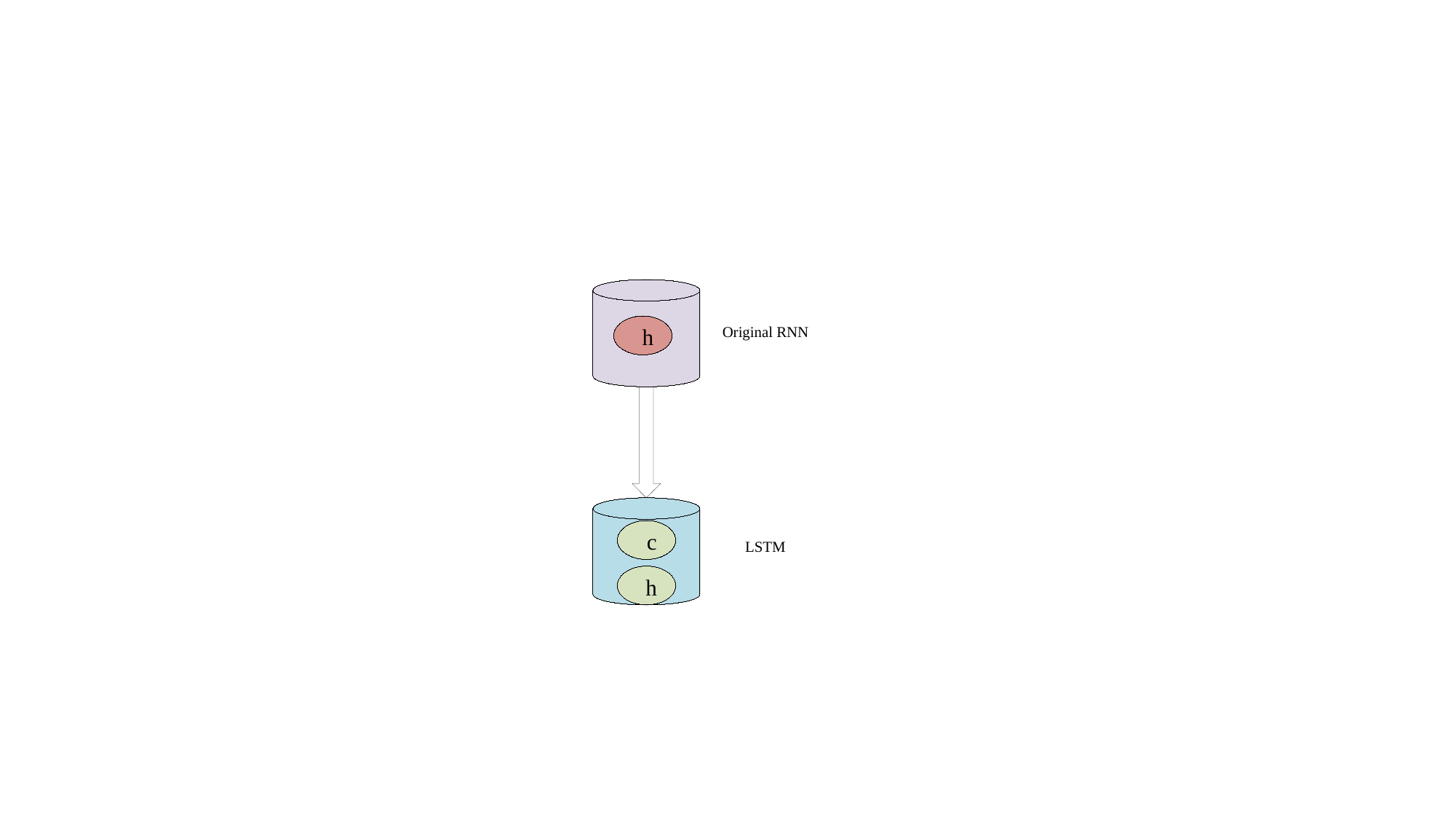

O
riginal RNN
h
c
LSTM
h

Supplement: S1 Data — (ZIP) [file pone.0285496.s001.zip › data/figure 5.pptx]

## Slide 1
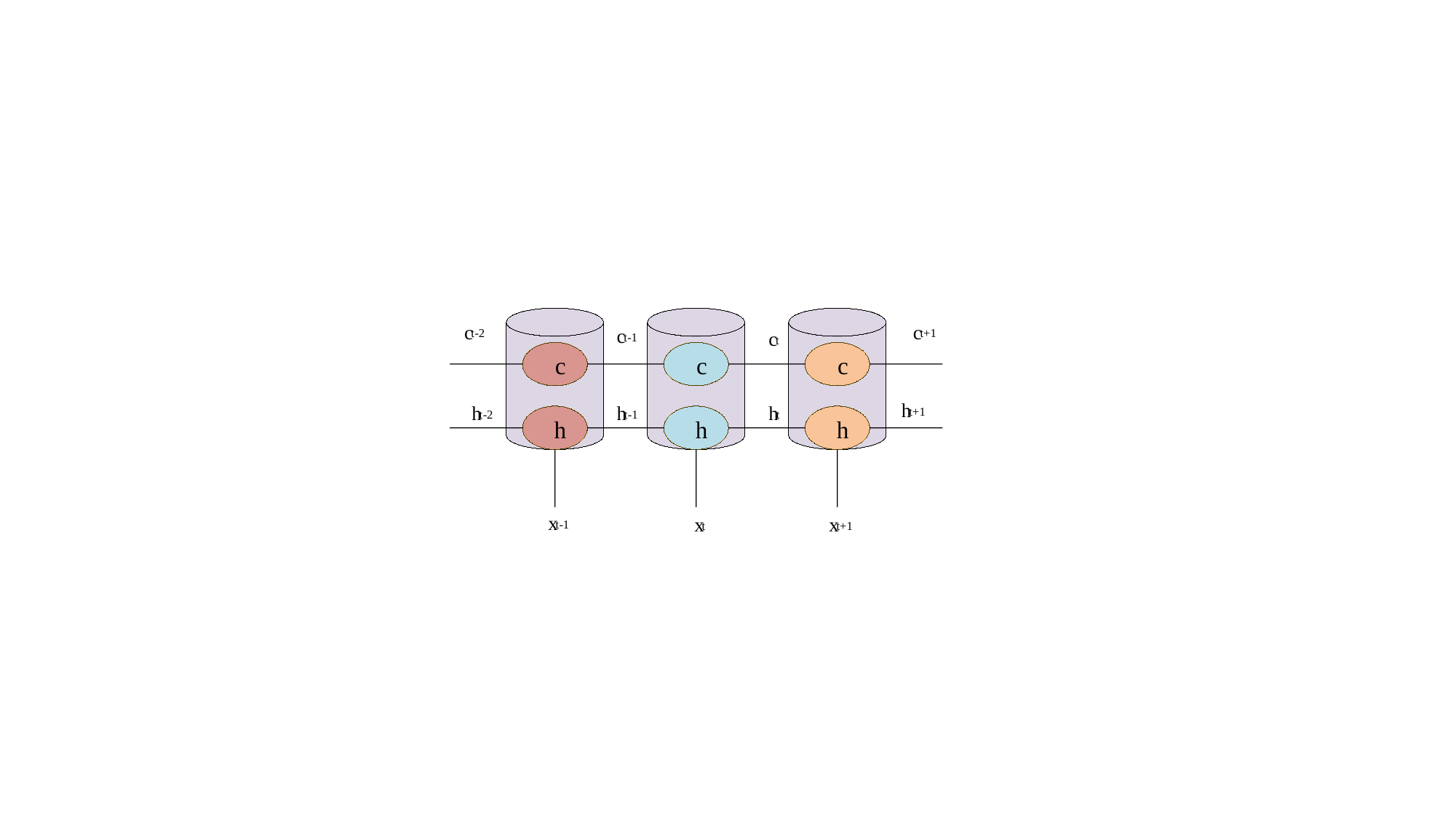

c
c
c
t
-
2
t+1
c
t
-
1
t
c
c
c
h
h
h
h
t+1
t
-
2
t
-
1
t
h
h
h
x
x
x
t
-
1
t
t+1

Supplement: S1 Data — (ZIP) [file pone.0285496.s001.zip › data/figure 6.pptx]
