# Supplementary material for: Intelligent auxiliary system for music performance under edge computing and long short-term recurrent neural networks
Source: PLoS One. 2023 May 8;18(5):e0285496. doi: 10.1371/journal.pone.0285496 (PMC10166492; doi:10.1371/journal.pone.0285496)
Supplement: Attachment — Submitted filename: Response to reviewers comments.docx [file pone.0285496.s003.docx]

### View Letter

| **Date:** | Apr 11 2023 02:57PM |
| --- | --- |
| **To:** | "Yi Wang" yvonnewang@ku.edu;wyyvonne_1021@outlook.com |
| **From:** | "PLOS ONE" plosone@plos.org |
| **Subject:** | PLOS ONE Decision: Revision required [PONE-D-22-29932R1] |

6. Review Comments to the Author

Reviewer #1: Authors have done necessary amendments in line with the recommendation of the reviewer. The manuscript in the current form may considered for the further phase of the editorial procedure.

Reply: Okay, thank you for your affirmation after careful reading. I am glad to enter the next stage and look forward to your reply.

Reviewer #2: 1) Parameter setting table is not suitable. Parameter name and values should be written in columns, in the first column parameter name and in the second one parameter values.

Reply: Okay, thank you for your feedback after carefully reading. I have adjusted the format of the parameter setting table, with the first column being the parameter name and the second column being the parameter value. Please refer to Table 1.
2) Author just added one table for LSTM. For the other methods such as lookback value and comparison of different synthesis models do not have table. They should also add tables to mentioned methods and then explain the result of each table.

Reply: OK, thank you for your comments after careful reading. I have added Table 3 below Section 3.2 to compare the loss function values of different cross domain sequence models under different look back values, to further reflect the advantages of the model proposed in this study.
